# Validity of very short answer versus single best answer questions for undergraduate assessment

**DOI:** 10.1186/s12909-016-0793-z

**Published:** 2016-10-13

**Authors:** Amir H. Sam, Saira Hameed, Joanne Harris, Karim Meeran

**Affiliations:** 1 Division of Diabetes, Endocrinology and Metabolism, Imperial College, London, UK; 2 Medical Education Research Unit, School of Medicine, Imperial College, London, UK

**Keywords:** Very short answer, Single best answer, Assessment, Testing, Validity, Reliability

## Abstract

**Background:**

Single Best Answer (SBA) questions are widely used in undergraduate and postgraduate medical examinations. Selection of the correct answer in SBA questions may be subject to cueing and therefore might not test the student’s knowledge. In contrast to this artificial construct, doctors are ultimately required to perform in a real-life setting that does not offer a list of choices. This professional competence can be tested using Short Answer Questions (SAQs), where the student writes the correct answer without prompting from the question. However, SAQs cannot easily be machine marked and are therefore not feasible as an instrument for testing a representative sample of the curriculum for a large number of candidates. We hypothesised that a novel assessment instrument consisting of very short answer (VSA) questions is a superior test of knowledge than assessment by SBA.

**Methods:**

We conducted a prospective pilot study on one cohort of 266 medical students sitting a formative examination. All students were assessed by both a novel assessment instrument consisting of VSAs and by SBA questions. Both instruments tested the same knowledge base. Using the filter function of Microsoft Excel, the range of answers provided for each VSA question was reviewed and correct answers accepted in less than two minutes. Examination results were compared between the two methods of assessment.

**Results:**

Students scored more highly in all fifteen SBA questions than in the VSA question format, despite both examinations requiring the same knowledge base.

**Conclusions:**

Valid assessment of undergraduate and postgraduate knowledge can be improved by the use of VSA questions. Such an approach will test nascent physician ability rather than ability to pass exams.

**Electronic supplementary material:**

The online version of this article (doi:10.1186/s12909-016-0793-z) contains supplementary material, which is available to authorized users.

## Background

Single Best Answer (SBA) questions are widely used in both undergraduate and postgraduate medical examinations. The typical format is a question stem describing a clinical vignette, followed by a lead in question about the described scenario such as the likely diagnosis or the next step in the management plan. The candidate is presented with a list of possible responses and asked to choose the single best answer.

SBA questions have become increasingly popular because they can test a wide range of topics with high *reliability* and are the ideal format for machine marking. They also have a definitive correct answer which is therefore not subject to interpretation on the part of the examiner.

However, the extent to which SBAs measure what they are intended to measure, that is their ‘*validity*,’ is subject to some debate. Identified shortcomings of SBAs include the notion that clinical medicine is often nuanced, making a *single* best answer inherently flawed. For example, we teach our students to form a differential diagnosis, but the ability to do this cannot, by the very nature of SBA questions, be assessed by this form of testing. Secondly, at the end of the history and physical examination, the doctor has to formulate a diagnosis and management plan based on information gathered rather than a ‘set of options’. Furthermore, the ability of an SBA to accurately test knowledge is affected by the quality of the wrong options (‘distractors’). Identifying four plausible distractors for SBAs is not always easy. This can be particularly challenging when assessing fundamental or core knowledge. If one or more options are implausible, the likelihood of students choosing the correct answer by chance increases. Thus the distractors themselves may enable students to get the correct answer without actually knowing the fact in question because candidates may arrive at the correct answer simply by eliminating all the other options.

Lastly, and perhaps most importantly, cueing is inherent to such a mode of assessment and with enough exam practice, trigger words or other recognised signposts contained within the question mean that ultimately, what is tested, is the candidate’s ability to pass exams rather than their vocational progress. It is therefore possible for candidates to get the answer correct even if their knowledge base is inadequate.

Within our own medical school, throughout the year groups, students undergo both formative and summative assessments by SBA testing. In order to address the shortcomings that we have identified in this model, we have developed a novel, machine marked examination in which students give a very short answer (VSA) which typically consists of three words or less. We hypothesised that VSAs would prove a superior test of inherent knowledge compared to SBA assessment.

## Methods

In a prospective pilot study, the performance of one cohort of 266 medical students in a formative examination was compared when the same knowledge was tested using either SBA or a novel, machine-marked VSA assessment. All students sat an online examination using a tablet computer. Questions were posed using a cloud-based tool and students provided answers on a tablet or smart phone. In the examination, 15 questions in the form of a clinical vignette (Table 1) were asked twice (Additional file 1: Supplementary methods).

**Table 1:** Blueprint of the questions asked in both very short answer (VSA) and single best answer (SBA) formats

Question	Specialty & Topic
1	Cardiology: diagnosis of left ventricular hypertrophy by voltage criteria on ECG
2	Cardiology: diagnosis of pericarditis
3	Respiratory Medicine: treatment of pneumonia
4	Gastroenterology: investigation of iron deficiency anaemia
5	Gastroenterology: clinical presentation of portal hypertension
6	Gastroenterology: diagnosis of hereditary haemorrhagic telangiecstasia
7	Endocrinology: investigation of hyponatraemia
8	Endocrinology: clinical presentation of thyrotoxicosis
9	Endocrinology: investigation of diabetic ketoacidosis
10	Haematology: diagnosis of haemolytic uraemic syndrome
11	Haematology: diagnosis of multiple myeloma
12	Nephrology: investigation of nephrotic syndrome
13	General surgery: small bowel obstruction on abdominal radiograph
14	Urology: investigation of suspected renal calculi
15	Breast Surgery: diagnosis of a breast mass

The first time the questions were asked, candidates were asked to type their response as a VSA, typically one to three words, to offer a diagnosis or a step in the management plan. The second time the questions were asked in an SBA format. We compared medical students’ performance between the questions in this format and the VSA format. In order to avoid the effect of cueing in subsequent questions, the exam was set in such a way that students could not return to previously answered questions.

**Fig. 1:**
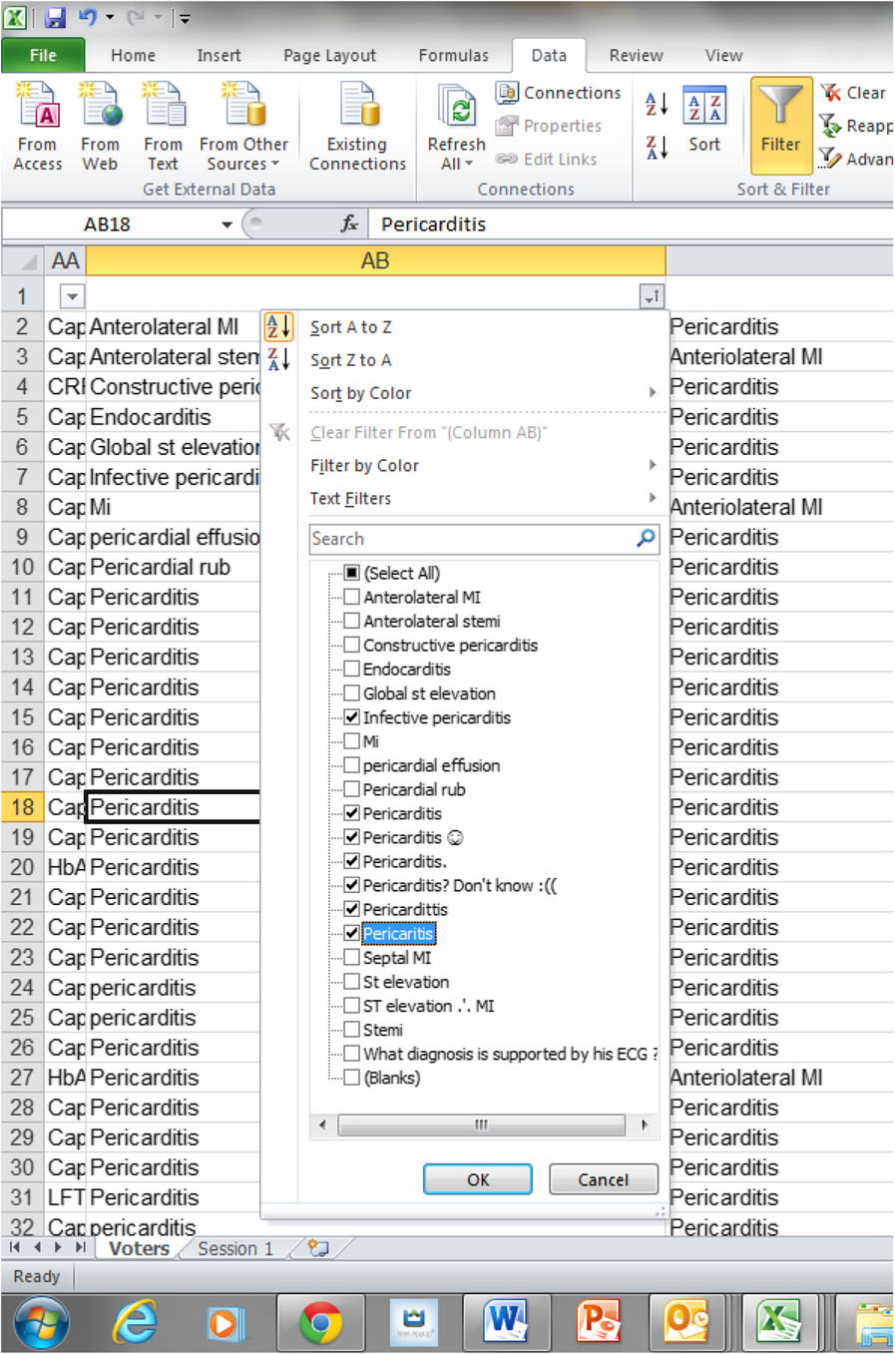
Marking method using Microsoft Excel and filter. In this example, candidates were given a clinical vignette and then asked to interpret the patient’s 12 lead electrocardiogram (ECG). There were 19 answers given by the students and seven variants of the correct answer (‘pericarditis’) were deemed acceptable as shown by the ticks in the filter

SBA responses were machine marked. The VSA data was exported into a Microsoft Excel spreadsheet. Using the ‘filter’ function in Microsoft Excel, we reviewed the range of answers for each question and assigned marks to acceptable answers. In this way, minor mis-spellings or alternative correct spellings could be rapidly marked as correct (Fig. 1). When several students wrote the same answer, this only appeared as one entry within the filter function to be marked. As a consequence, the maximum time spent on marking each question for 266 students was two minutes.

The McNemar’s test was used to compare the students’ responses to VSA and SBA questions.

## Results

**Fig. 2:**
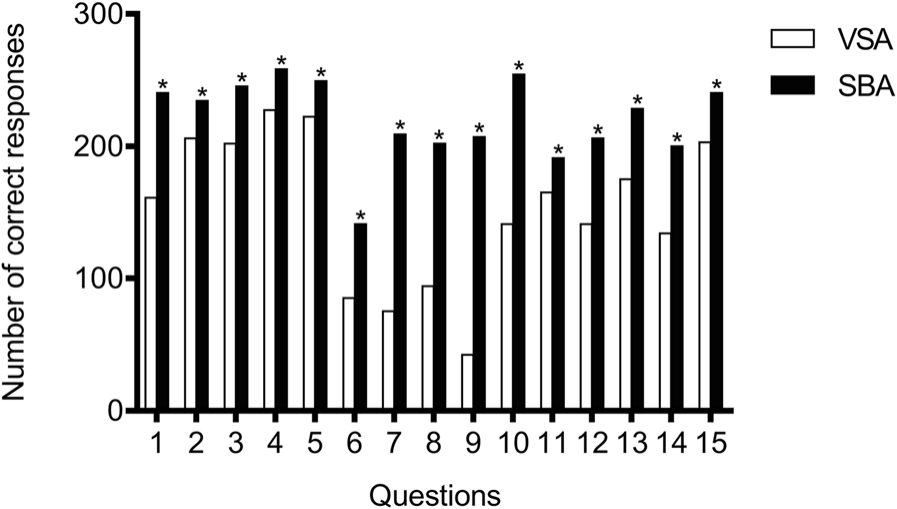
Number of correct responses given for questions asked in two formats: single best answer (SBA) and very short answer (VSA). Medical students were given a clinical vignette and asked about clinical presentations, investigations and treatment of the patient. In the VSA format, students were asked to type the answer (one to three words). In the SBA format, students chose the correct answer from a list. In all questions, more students got the correct answer when given a list to choose from in the SBA format than when asked to type the answer in the VSA assessment (* *p* < 0.01)

There was a statistically significant difference in the proportions of correct/incorrect answers to the VSA and SBA formats in all 15 questions (*p* < 0.01). Figure 2 shows the numbers of students who got each question correct, either as a VSA or an SBA. In all questions, more students got the correct answer when given a list to choose from in the SBA format than when asked to type words in the VSA examination. For example, when asked about the abnormality on a blood film from a patient with haemolytic ureamic syndrome, only 141 students offered the correct VSA. However a further 113 students who didn’t know this, guessed it correctly in the SBA version of the question.

## Discussion

The high scoring of the SBA questions in comparison to the VSA format is a cause for concern for those involved in the training of fit for purpose doctors of tomorrow. Despite the same knowledge being tested, the ability of some students to score in the SBA but not in the VSA examination demonstrates that reliance on assessment by SBA can foster the learning of association and superficial understanding to pass exams.

Assessment is well known to drive learning [1] but it only drives learning that will improve performance in that type of assessment. Studying exam technique rather than the subject is a well-known phenomenon amongst candidates [2]. In the past 20 years there has been an emphasis on the use of tools such as the SBA that offer high reliability in medical school assessment [3]. The replacement of marking of written exams with machine marked SBAs has resulted in students engaging in extensive and strategic practice in that exam technique. Students who practise large numbers of past questions can become adept at choosing the correct option from a list without an in-depth understanding of the topic. While practising exam questions can increase knowledge, the use of cues to exclude distractors is an important skill in exam technique with SBAs. This technique improves performance in the assessment, but does not enhance the student’s ability to make a diagnosis in clinical situations. Students who choose the correct option in SBAs may be unable to answer a question on the same topic when asked verbally by a teacher who does not present them with options. In clinical practice a patient will certainly not provide a list of possible options. We are thus sacrificing validity for reliability.

One of the key competencies for the junior doctors is to be able to recall the correct diagnosis or test in a range of clinical scenarios. In a question about a patient with suspected diabetic ketoacidosis (DKA), only 42 students offered to test capillary or urinary ketones, whereas when the same question was posed in the SBA format, another 165 students chose the correct answer. We expect our junior doctors to recall this important immediate step in the management of an unwell patient with suspected DKA. Our findings therefore suggest that assessment by VSA questions may offer added value in testing this competency.

An ideal assessment will encourage deep learning rather than recognition of the most plausible answer from a list of options. Indeed tests that require students to construct an answer appear to produce better memory than tests that require students to recognise an answer [4].

In contrast to SBA, our pilot study has demonstrated that to correctly answer a VSA, students need to be able to generate the piece of knowledge in the absence of cues, an approach that is more representative of real-life medical practice.

Our increasing reliance on assessment by SBA is partly an issue of marking manpower. SBAs can be marked by a machine, making them a highly efficient way to generate a score for a large number of medical students. Any other form of written assessment requires a significant investment of time by faculty members to read and mark the examination. However, our novel machine marked VSA tested knowledge and understanding but each question could still be marked in two minutes or less for the entire cohort. The use of three or less words allowed for a stringent marking scheme, thus eliminating the inter-marker subjectivity which can be a problem in other forms of free text examination.

It must be emphasised that there is no single ideal mode of assessment and that the best way to achieve better reliability and validity is by broad sampling of the curriculum using a variety of assessment methods. This is a pilot study and further research should evaluate the reliability, validity, educational impact and acceptability of the VSA question format.

## Conclusions

This pilot study highlights the need to develop machine marked assessment tools that will test learning and understanding rather than exam technique proficiency. This study suggests that students are less likely to generate a correct answer when asked to articulate a response to a clinical vignette than when they have to pick an answer from a list of options. This therefore raises the possibility that the VSA is a valid test of a student’s knowledge and correlation of VSA marks with other modes of assessment should be investigated in future research. Future examinations may be enhanced by the introduction of VSAs, which could add an important dimension to assessments in clinical medicine.

## Additional file

Additional file 1: Supplementary Methods. Questions used in Single Best Answer (SBA) and Very Short Answer (VSA) formats. (DOCX 22 kb)

## Abbreviations

ECG: Electrocardiogram
SAQs: Short answer questions
SBA: Single best answer
VSA: Very short answer
