# Supplementary material for: Validity of very short answer versus single best answer questions for undergraduate assessment
Source: BMC Med Educ. 2016 Oct 13;16:266. doi: 10.1186/s12909-016-0793-z (PMC5064885; doi:10.1186/s12909-016-0793-z)
Supplement: Additional file 1: — Supplementary Methods. Questions used in Single Best Answer (SBA) and Very Short Answer (VSA) formats. (DOCX 22 kb) [file 12909_2016_793_MOESM1_ESM.docx]

**Supplementary Methods**

**Validity of very short answer versus single best answer questions for undergraduate assessment**

Amir H Sam^1, 2^ MRCP, Saira Hameed^1^ MRCP, Joanne Harris^2^ MRCP, Karim Meeran^1, 2^ FRCP, FRCPath

1. Division of Diabetes, Endocrinology and Metabolism, Imperial College London, UK

2. Medical Education Research Unit, School of Medicine, Imperial College London, UK

Amir H Sam a.sam@imperial.ac.uk; Saira Hameed s.hameed@imperial.ac.uk; Joanne Harris joanne.harris@imperial.ac.uk; Karim Meeran k.meeran@imperial.ac.uk

**Corresponding author:** Professor Karim Meeran (k.meeran@imperial.ac.uk)

**Questions used in Single Best Answer (SBA) and Very Short Answer (VSA) formats**

**Question 1**

**VSA**

A 60 year old woman presents with collapse. Her blood pressure is 120/70 mmHg and there is no postural drop. On auscultation there is an ejection systolic murmur. What does the ECG show?

**Acceptable answers:** left ventricular hypertrophy, LVH

**SBA** (correct answer in bold)

A 60 year old woman presents with collapse. Her blood pressure is 120/70 mmHg and there is no postural drop. On auscultation there is an ejection systolic murmur. What does the ECG show?

1. Left atrial hypertrophy
2. **Left ventricular hypertrophy**
3. Normal ECG
4. Right atrial hypertrophy
5. Right ventricular hypertrophy

**Question 2**

**VSA**

A 26 year old man has chest pain. He smokes 5 cigarettes per day. On auscultation there is a ‘scratching sound’. What diagnosis is supported by his ECG?

**Acceptable answers:** pericarditis, infective pericarditis

**SBA** (correct answer in bold)

A 26 year old man has chest pain. He smokes 5 cigarettes per day. On auscultation there is a ‘scratching sound’. What diagnosis is supported by his ECG?

1. Anterolateral MI
2. Inferior MI
3. NSTEMI
4. **Pericarditis**
5. Posterior MI

**Question 3**

**VSA**

A 45 year old man has cough and breathlessness. He reports a recent travel. On examination of the chest he has coarse crepitations and bronchial breathing. His blood tests show hyponatraemia and deranged liver function tests. He has no drug allergies. What antibiotic would you prescribe in addition to amoxicillin?

**Acceptable answers:** A macrolide antibiotic such as clarithromycin or erythromycin (according to curriculum and local protocols).

**SBA** (correct answer in bold)

A 45 year old man has cough and breathlessness. He reports a recent travel. On examination of the chest he has coarse crepitations and bronchial breathing. His blood tests show hyponatraemia and deranged liver function tests. He has no drug allergies. What antibiotic would you prescribe in addition to amoxicillin?

1. Cefuroxime
2. **Clarithromycin**
3. Co-amoxiclav
4. Tazocin
5. Vancomycin

**Question 4**

**VSA**

A 50 year old man has dyspepsia and weight loss. His full blood count shows: Hb 70 g/L and MCV 70 fl. What is the next most appropriate investigation?

**Acceptable answers:** Upper GI endoscopy, endoscopy, OGD, oesophago-gastro-duodenostomy, gastroscopy

**SBA** (correct answer in bold)

A 50 year old man has dyspepsia and weight loss. His full blood count shows: Hb 70 g/L and MCV 70 fl. What is the next most appropriate investigation?

1. Abdominal CT
2. Abdominal USS
3. Colonoscopy
4. Erect Chest X-ray
5. **OGD (gastroscopy)**

**Question 5**

**VSA**

What is the name of the clinical sign shown in this picture?

**Acceptable answer:** caput medusae

**SBA** (correct answer in bold)

What is the name of the clinical sign shown in this picture?

1. **Caput medusae**
2. Grey Turner
3. Troisier’s sign
4. Trousseau’s sign
5. Virchow’s node

**Question 6**

**VSA**

A 30 year old man has recurrent gastrointestinal and nose bleeds. His face is shown in the picture below. What is the diagnosis?

**Acceptable answers:** Hereditary Haemorrhagic Telangiecstasia, Osler-Weber-Rendu Disease

**SBA** (correct answer in bold)

A 30 year old man has recurrent gastrointestinal and nose bleeds. His face is shown in the picture below. What is the diagnosis?

1. Acromegaly
2. Cirrhosis
3. **Hereditary Haemorrhagic Telangiecstasia**
4. Peutz-Jegher syndrome
5. Systemic sclerosis

**Question 7**

**VSA**

A 60 year old man has confusion and a cough. On examination there is no postural hypotension. His blood tests show: sodium 120 mmol/L and potassium 4 mmol/L. His thyroid function test and short Synacthen test are normal. His urine sodium is 30mmol/L and his urine osmolality is 400 mmol/kg. What is the next most appropriate investigation?

**Acceptable answers:** CXR, chest X-ray, chest radiograph

**SBA** (correct answer in bold)

A 60 year old man has confusion and a cough. On examination there is no postural hypotension. His blood tests show: sodium 120 mmol/L and potassium 4 mmol/L. His thyroid function test and short Synacthen test are normal. His urine sodium is 30mmol/L and his urine osmolality is 400 mmol/kg. What is the next most appropriate investigation?

1. Brain MRI
2. CT Abdomen
3. **Chest X-ray**
4. Lung function tests
5. OGD

**Question 8**

**VSA**

A 35 year old man has sweating and weight loss. What is the name of the clinical sign shown in this picture?

**Acceptable answer:** Onycholysis

**SBA** (correct answer in bold)

A 35 year old man has sweating and weight loss. What is the name of the clinical sign shown in this picture?

1. Beau’s lines
2. Koilonychia
3. Leukonychia
4. Nail pitting
5. **Onycholysis**

**Question 9**

**VSA**

A 20 year old woman has abdominal pain and vomiting. She has type 1 diabetes. Her capillary blood glucose is 20mmol/L. What is the next most appropriate investigation?

**Acceptable answers:** ketones, urinary ketones, blood ketones, capillary ketones

**SBA** (correct answer in bold)

A 20 year old woman has abdominal pain and vomiting. She has type 1 diabetes. Her capillary blood glucose is 20mmol/L. What is the next most appropriate investigation?

1. **Capillary ketones**
2. CRP
3. FBC
4. HbA1c
5. LFT

**Question 10**

**VSA**

A 20 year old boy has diarrhoea and malaise. His blood tests show: Hb 70 g/L, Cr 300 μmol/L. What do the arrows on this blood film show?

**Acceptable answers:** Schistocytes, red cell fragments

**SBA** (correct answer in bold)

A 20 year old boy has diarrhoea and malaise. His blood tests show: Hb 70 g/L, Cr 300 μmol/L. What does the arrow on this blood film show?

1. Codocytes (target cells)
2. Elliptocytes
3. Lymphocytes
4. **Schistocytes (red cell fragments)**
5. Spherocytes

**Question 11**

**VSA**

A 50 year old man has backache. His blood tests show hypercalcaemia, low PTH and normal ALP. What is the most likely diagnosis?

**Acceptable answers:** Multiple myeloma, myeloma

**SBA** (correct answer in bold)

A 50 year old man has backache. His blood tests show hypercalcaemia, low PTH and normal ALP. What is the most likely diagnosis?

1. Bone metastases
2. **Multiple myeloma**
3. Osteoporosis
4. Primary hyperparathyroidism
5. Secondary hyperparathyroidism

**Question 12**

**VSA**

A 35 year old woman has ankle oedema. Her echocardiogram is normal. Her blood tests show normal renal function, ALT, AST and ALP. Her albumin is 15g/L. What is the next most appropriate investigation?

**Acceptable answers:** urinalysis, urine protein, urine dipstick

**SBA** (correct answer in bold)

A 35 year old woman has ankle oedema. Her echocardiogram is normal. Her blood tests show normal renal function, ALT, AST and ALP. Her albumin is 15g/L. What is the next most appropriate investigation?

1. Coronary angiogram
2. Renal USS
3. Repeat LFT
4. Troponin
5. **Urinalysis**

**Question 13**

**VSA**

What does the arrow show in this abdominal x-ray?

**Acceptable answers:** Valvulae conniventes

**SBA** (correct answer in bold)

What does the arrow show in this abdominal x-ray?

1. Adhesions
2. Haustra
3. Large bowel
4. Stomach
5. **Valvulae conniventes**

**Question 14**

**VSA**

A 40 year old man has loin pain. He has a normal CRP. Urinalysis shows blood ++. What is the next most appropriate investigation?

**Acceptable answers:** CT KUB, CT kidneys/ureters/bladder, CT renal tract

**SBA** (correct answer in bold)

A 40 year old man has loin pain. He has a normal CRP. Urinalysis shows blood ++. What is the next most appropriate investigation?

1. Abdominal USS
2. Abdominal X-ray
3. **CT KUB**
4. CT with contrast
5. MR Angiogram

**Question 15**

**VSA**

A 23 year old woman has a 1 cm smooth mobile breast mass. What is the most likely diagnosis?

**Acceptable answers:** fibroadenoma

**SBA** (correct answer in bold)

A 23 year old woman has a 1 cm smooth mobile breast mass. What is the most likely diagnosis?

A. Basal cell carcinoma
B. Ductal carcinoma
C. Fat necrosis
**D. Fibroadenoma**
E. Galactocoele
